# Identifying temporal eating patterns: a comparison of latent class analysis and dynamic time warping-based cluster analysis

**DOI:** 10.1016/j.ajcnut.2026.101317

**Published:** 2026-04-15

**Authors:** Beshada R Jima, Rebecca M Leech, David W Dunstan, Abbas Z Kouzani, Sarah A McNaughton

**Affiliations:** 1 Institute for Physical Activity and Nutrition, School of Exercise and Nutrition Sciences, Deakin University, Burwood, Victoria, Australia; 2 Baker Heart & Diabetes Institute, Melbourne, Victoria, Australia; 3 Deakin University, School of Engineering, Geelong, Victoria, Australia; 4 Health and Well-Being Centre for Research Innovation, School of Human Movement and Nutrition Sciences, University of Queensland, St Lucia, Queensland, Australia; 5 School of Exercise and Nutrition Sciences, Deakin University, Geelong, Victoria, Australia

**Keywords:** temporal eating patterns, diet quality, obesity, latent class analysis, dynamic time warping, cluster analysis

## Abstract

**Background:**

Temporal eating patterns (TEPs) are associated with diet quality and obesity, although inconsistencies exist because of variations in methods and input variables. Direct comparisons of analytical approaches for deriving TEPs are rare.

**Objectives:**

The aim of this study was to compare latent class analysis (LCA) and modified dynamic time warping (MDTW)–based cluster analysis for deriving TEPs and examine their associations with diet quality and obesity.

**Methods:**

This cross-sectional study included 672 adults (18–65 y) in Victoria, Australia, from the “EveryDayLife” survey (2017–2020). Participants completed a 1- to 7-d food diary via the “FoodNow” app. LCA used hourly presence/absence of eating occasions (EOs), whereas MDTW-based clustering used hourly energy intake (EI) input variables. Methods were compared using patterns visualization, membership overlap, kappa statistics, adjusted *R*^2^, and AUC.

**Results:**

Both methods identified 3 distinct TEPs. Class 1/Cluster 1 had peaks during 07:00–09:00, 12:00, and 18:00–19:00 h (“conventional” pattern). Class 2/Cluster 2 had later peaks in EOs or EI (after 13:00 h). Class 3/Cluster 3 showed modest, evenly spaced EOs or EI concentrated earlier in the day. Membership overlap between similar TEPs was 56.2%–73.1%, with fair agreement (κ = 0.38, *P* < 0.001). Class 1/Cluster 1 showed higher diet quality than Class 2/Cluster 2, respectively, whereas no significant associations were observed with BMI. LCA explained slightly more variance in diet quality (6% compared with 4%) compared with MDTW-based clustering, with a similar proportion observed for BMI (∼13%). The AUCs for discriminating high diet quality (LCA: 0.635 compared with MDTW: 0.616; *P* = 0.565) and obesity (LCA: 0.758 compared with MDTW: 0.756; *P* = 0.934) were not significantly different between the 2 methods.

**Conclusions:**

LCA and MDTW-based clustering identified comparable yet noninterchangeable TEPs, suggesting both approaches are suitable for deriving TEPs.

## Introduction

The prevalence of obesity has increased worldwide and remains a major global health concern [1]. In Australia, ∼32% adults were affected by obesity in 2022 [2]. Obesity is both a consequence of, and a contributor to metabolic syndrome and type 2 diabetes [3]. To better understand dietary determinants of obesity, recent research has focused on temporal eating patterns (TEPs), which refer to the timing, frequency, and sequence of eating occasions (EOs) or energy intake (EI) throughout the day [4]. The timing of food intake modulates peripheral clocks in organs, including the liver, muscle, and pancreas [5], whereas the central clock in the brain’s suprachiasmatic nucleus, entrained by the light–dark cycle, regulates physiological functions, including nutrient absorption and metabolism [6]. The disruptions in this central-peripheral clock coordination (circadian misalignment) may impair metabolism and increase obesity and cardiometabolic risk [7]. Importantly, people consume EOs (meals or snacks) at specific times, and intake at 1 EO may affect subsequent intake and total daily consumption, which may influence diet quality and health [8]. Therefore, understanding TEPs may help to identify patterns that may be associated with adverse health outcomes.

Latent class analysis (LCA) [[9], [10], [11]] and cluster analysis [[12], [13], [14], [15], [16], [17], [18]] are the commonly used approaches for deriving TEPs. LCA identify latent classes based on the probability of shared characteristics using categorical input data, whereas cluster analysis, particularly when combined with dynamic time warping (DTW), aligns complex temporal data. DTW measures similarity between sequences through an optimal warping path [19,20]. A modified version of DTW (MDTW) addresses traditional DTW’s excessive flexibility by constraining alignments of time points, which improves the identification of meaningful temporal patterns when combined with cluster analysis [21].

Evidence that examined the association of TEPs with their diet quality and obesity remains limited and inconsistent [10,11,[13], [14], [15], [16],^18^,^22^]. Eicher-Miller et al. [13] used DTW-based cluster analysis and identified 4 TEPs among US adults. They reported that a pattern with moderate, evenly spaced EI throughout the day was associated with more favorable diet quality. In Australian adults, Leech et al. [22] found higher diet quality among females following “Conventional” type pattern and among males with a “Later lunch” type pattern using LCA. Similarly, Song et al. [10] reported more favorable diet quality among individuals following a “Noon dominant” pattern using LCA.

Studies that examine the association between TEPs and obesity have also shown mixed findings. Most studies have reported that a “Grazing” type pattern with frequent EOs across the day was associated with higher BMI and greater odds of obesity compared with “Conventional” type patterns [[13], [14], [15], [16],^18^]. However, Jayedi et al. [11] reported that the “Grazing” type pattern was associated with lower odds of obesity among females. Furthermore, TEPs with higher EI at midday and in the evening have been associated with higher BMI [14,15,18].

The inconsistencies across studies may be due to variations in analytical methods, input variables, and time intervals (e.g., minute, hour, or 4-h blocks), which may result in nuances for the identified patterns. Therefore, determining suitable methods for deriving TEPs is a critical first step to advance the understanding of how TEPs associate with health outcomes. Determining the most suitable methods may also enhance pattern interpretability and clarify how methodological choices affect observed associations with diet quality and obesity. Such insights are important for informing interventions aimed at reducing chronic disease-related morbidity. Accordingly, this study compared LCA and MDTW-based cluster analysis for deriving TEPs and examined their associations with overall diet quality and obesity among Australian adults.

## Methods

### Participants and procedures

This cross-sectional study utilized data from the “EveryDayLife” survey (2017–2020), whose collection was funded by the Australian Research Council (DP170100544), which aimed to examine temporal patterns of eating, physical activity, and sleep in relation to mood among adults aged 18–65 y in Victoria, Australia. Participants were recruited through targeted advertisements via social media (e.g., Facebook and Twitter), local newspapers, and posters placed around venues where the target population frequents (e.g., sporting clubs, shopping centers, and universities). After providing informed consent, participants accessed the online Qualtrics survey and completed a food diary using the “FoodNow” smartphone app. The online Qualtrics survey collects data on sociodemographic characteristics, health indicators, physical activity, sedentary behavior, and sleep. Reminder messages were sent to encourage participation, and a $20 voucher was provided upon diary completion. Ethics approval was granted by Deakin University Human Research Ethics Committee (reference: 2017-211), and the study followed STROBE-nut reporting guidelines [23].

### Dietary assessment

Dietary intake was recorded using the “FoodNow” smartphone app [24,25]. Participants recorded all foods and beverages consumed and time eating started, including food items’ images and completed a series of questions detailing the type and quantity of items and the type of EO (e.g., breakfast and lunch). Push notifications reminded participants to log intake if no entry was made within 3 h during 09:00–21:00 h. The following day, participants reported supplement use, whether intake was usual, and any missed or unrecorded occasions. The “FoodNow” app was piloted and validated in adults and showed good agreement with energy expenditure (EE) measured by SenseWear Armband (BodyMedia Inc) [24,26].

The reported food and beverage items were coded using the Australian Food, Supplement, and Nutrient Database (AUSNUT 2011–2013) by trained nutritionists [27]. Coding decisions were guided by recorded images and responses to a series of questions to accurately determine the type and quantity of each food item. Accuracy of coding was verified through a duplicate review process, and any discrepancies were resolved in consultation with a dietitian until consensus was reached. Dietary data cleaning during coding involved verifying complete nutrient imputation for all coded foods, performing range checks for key macro and micronutrients at the individual day level, and reviewing values exceeding ± 3SD to identify potential data entry or reporting errors.

Dietary data were collected for each participant over a period of 1–7 d. Three quarters of participants provided complete 7-d dietary records (*n* = 506, 75.3%). The remaining participants contributed dietary data for fewer days, including 1 d (*n* = 46, 6.9%), 2 d (*n* = 45, 6.7%), 3 d (*n* = 20, 3.0%), 4 d (*n* = 12, 1.8%), 5 d (*n* = 14, 2.1%), and 6 d (*n* = 29, 4.2%). Four recorded days were removed from the dataset because the participants reported zero EI on those days. Total daily EI, food group consumption, eating patterns, and diet quality scores were calculated by averaging dietary data across all recorded days. Hourly EI, used as an input variable for deriving TEPs, was calculated by averaging the energy consumed from foods and beverages at each EO that occurred within the same hour across all recorded days.

### EOs

EO was defined as any food or drink event providing EI of ≥210 kJ, occurring ≥15 min apart from prior or subsequent EO [9]. The mean EO frequency was calculated. Participants reported the types of EOs, with breakfast, brunch, lunch, dinner, and supper categorized as meals, whereas snack breaks, teas, beverages, and other occasions classified as snacks [9]. Extended eating within 15 min of a prior meal or snack was grouped with the preceding meal or snack, respectively [9]. The mean frequencies of meals and snacks and their respective contributions to total EI (i.e., eating patterns) were also calculated.

### Diet quality

Diet quality was assessed using the Dietary Guideline Index (DGI), which reflects adherence to the 2013 Australian Dietary Guidelines (ADG) [28]. The DGI includes 13 components: 7 components assess adequacy (e.g., variety, intake of vegetables, fruits, cereals, dairy, lean meats, and fluids), and 6 components assess moderation (e.g., discretionary foods, saturated fat, added sugar, alcohol, salt, and unsaturated fat) [29,30]. For this study, the added salt component was excluded due to data not being captured in the “FoodNow” app, resulting in a total DGI score range of 0–120. DGI score component cutoffs were based on age- and sex-specific ADG recommendations. Participants were grouped by DGI score into 3 categories: *1*) high diet quality: those in the top tertile (72.6–110.4), *2*) medium diet quality: those in the middle tertile (63.3–72.5), and *3*) low diet quality: those in the bottom tertile (34.4–63.2).

### Anthropometry

Anthropometric measures, including height and weight, were self-reported, and BMI was calculated using a previously established correction equation that has been used in previous studies [[31], [32], [33]]. Evidence indicates that factors such as social pressure and self-esteem may contribute to the under-reporting of weight and over-reporting of height [34]. Participants were classified as “with obesity” (BMI ≥30 kg/m^2^) or “without obesity” (BMI <30 kg/m^2^), as the overweight threshold (BMI ≥25 kg/m^2^) is more likely to result in misclassification when based on self-reported data [35]. This classification also aligns with most studies examining TEPs and obesity, which support comparability of findings [11,15].

### Energy misreporting

Evidence indicates that EI is often misreported, which affects the estimates of diet quality and its associations with obesity [36,37]. Under-reporting has also been linked to irregular meal patterns [38] and fewer EOs [39]. To account for this, the current analysis adjusted for energy misreporting, which was assessed using the ratio of total daily EI to the estimated EE [40]. EE was calculated with a validated equation incorporating age, weight, height, and physical activity level according to the Institute of Medicine [41]. Evidence suggests that most Australian adults do not meet the Physical Activity Guidelines and predominantly engage in sedentary or light-intensity activity [42,43]. Therefore, a low physical activity level (≤1.4) was applied in the estimation of EE because of the absence of objective physical activity data, consistent with previous studies [22,40]. Participants were classified as plausible, under-reporters, or over-reporters of EI based on established SD cutoff calculations for the EI-to-EE ratio, which accounted for the coefficient of variance for mean daily EI and estimated EE [22,40].

### Covariates

During the online questionnaire, age was recorded as a continuous variable, and sex as male, female, or indeterminate/intersex/unspecified; however, only male and female categories were included in this study, as serving cutoffs for diet quality calculation were not available for indeterminate, intersex, or unspecified sex. Education level was classified as “year 12 or less,” “still studying for first qualification,” “certificate,” and “tertiary” [25]. Country of birth was grouped as “Australia” and “outside Australia” [9]. Relationship status was categorized as “married/living together,” “in a relationship but not living together,” and “not in a relationship.” Occupation was classified into 4 groups: “manager/professional,” “technician/trade worker/machinery operator/driver/laborer,” “community worker/personal service worker/clerical worker/administrative worker/sales worker,” and “no paid job/student/other.” Household income referred to gross weekly equivalized income and was split into quartiles from lowest to highest 25%. Usual weekly work hours were recorded, and work patterns were classified as “shift” or “nonshift.” Smoking status was categorized as “nonsmoker” and “past/current smoker,” based on evidence of similar dietary behaviors [44]. Alcohol drinking was classified as a binary variable (≤4 or >4 standard drinks/d) based on national guidelines for reducing long-term alcohol-related harm in adults [45]. Physical activity was assessed using the International Physical Activity Questionnaire–Short Form [46] and categorized as “met-guideline” and “unmet-guideline” based on national recommendations (i.e., ≥150 min of moderate, ≥75 min of vigorous activity, and ≥5 sessions/wk) [47].

### Analytic sample

**FIGURE 1:**
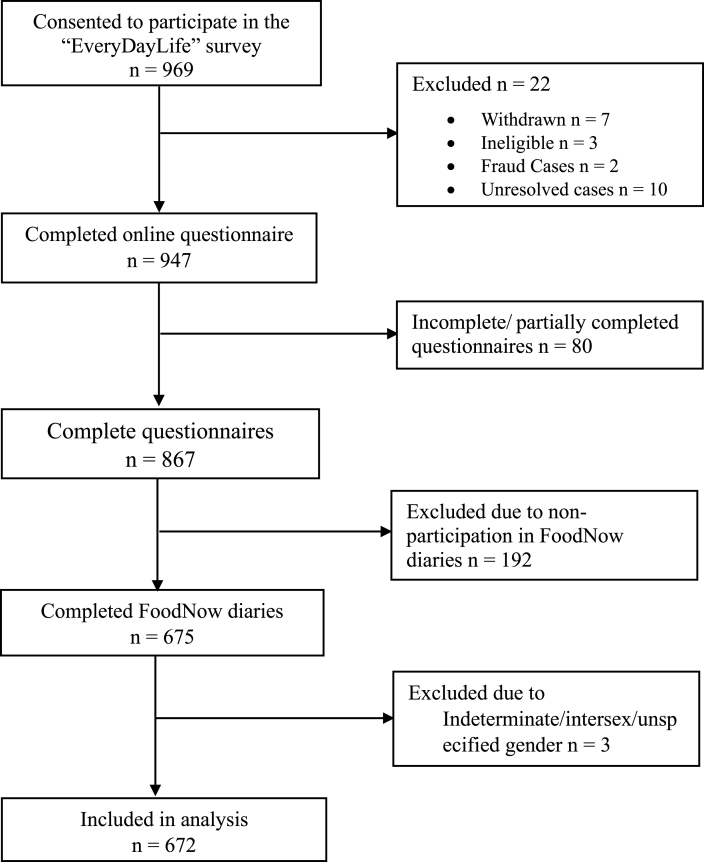
Flow of the participants.

The eligibility criteria for survey participation included being aged 18–65 y, residing in Victoria state, owning a smartphone purchased in Australia, not being pregnant or breastfeeding, and having English as the primary language spoken at home. Initially, a total of 969 participants consented to participate in the survey. Of these, 22 were excluded due to withdrawal (*n* = 7), ineligibility (*n* = 3), fraud (*n* = 2), or unresolved cases (*n* = 10). A further 80 participants were excluded because of incomplete or partially completed questionnaires, and 192 were excluded due to nonparticipation in the FoodNow dietary assessment. Additionally, 3 participants (*n* = 3) were excluded due to indeterminate, intersex, or unspecified sex. Shift workers were retained in the analysis because only a subset of participants (*n* = 346) reported their usual work patterns, and among these, only a small number (*n* = 2) were permanent night shift workers. The final analytical sample comprised 672 participants (Figure 1).

### Statistical analysis

#### LCA

LCA is a statistical method used to identify hidden or latent patterns within observable indicator variables, with each individual in the sample assigned to a distinct, mutually exclusive class [48]. LCA was performed using M-Plus software (Version 8.11; Muthén & Muthén), employing a binary variable [EI ≥210 kJ (50 kcal) occurred at each hour or not]. The number of latent classes was determined based on 3 key criteria: *1*) model fit indices, such as the Akaike Information Criterion and Bayesian Information Criterion, where lower values suggested a better model fit; *2*) the Lo-Mendell–Rubin–Adjusted Likelihood Ratio Test and Bootstrap Likelihood Ratio Test, which compared models with *k* latent classes to those with *k*-1 latent classes; and *3*) the interpretability of the resulting classes [49]. The model fit indices supported a 3-class model (Supplementary Table 1). Additionally, the mean conditional probabilities for the most likely latent class membership of 3-class model were high, ranging from 0.84 to 0.96, indicating good classification quality (Supplementary Table 2), based on the conditional probability of each participant (Supplementary File 2).

Latent profile analysis (LPA), a continuous-variable analog of LCA, was conducted using hourly EI to compare patterns using similar input variables with MDTW. However, LPA did not produce meaningful patterns, specifically with regard to pattern size (see Supplementary Figure 1).

#### MDTW-based cluster analysis

DTW is a similarity measure used to compare time series that may differ in length or be misaligned in time by identifying an optimal warping path [19]. This similarity measure is commonly used in speech recognition and time series pattern detection [20]. DTW aligns sequences by stretching or compressing the time axis and generates a distance matrix that quantifies pairwise similarity, which can subsequently be used for cluster analyses. Another comparative method (such as Euclidean distance) performs well with aligned time points but fails to account for individual variations in meal timing and is therefore less suitable for identifying TEPs [12]. Standard DTW also faces limitations when EOs are missing or skipped, resulting in unmatched events and less accurate distance estimates.

MDTW addresses misalignments caused by variations in wake-up times, meal timing, and the number of EOs across individuals [21], resulting in a more appropriate distance matrix for cluster analysis and potentially more interpretable patterns. Unlike standard *k*-means, kernel *k*-means clustering considers both the timing and the distribution of EI. Integrating MDTW with kernel *k*-means improves clustering by aligning time series while capturing similarities in both meal timing and energy distribution, which allows the identification of more meaningful and interpretable TEPs.

In this study, MDTW combined with kernel *k*-means cluster analysis was implemented using Python software (version 3.13.5: Python Software Foundation) for identifying TEPs based on continuous hourly mean EI (the Python code is available in Supplementary File 3). A local constraint was applied to ensure appropriate alignment between an individual’s hourly EI and that of others, which allowed matches only when time points fell within a 3-h window (Sakoe–Chiba band). This 3-h window constraint preserves the hourly resolution of EI while allowing limited temporal flexibility to accommodate small shifts in intake timing because of behavioral, social, and physiological factors such as sleep wake cycles, work schedules, commuting times, etc. This flexibility is important for deriving meaningful TEPs from a dietary pattern perspective. The optimal number of clusters was determined using a combination of internal and external criteria. Internal criteria include the Silhouette and Dunn indices, where higher values indicate better cluster solutions. External criteria included visual inspection of the patterns and evaluation of differences in both the timing and distribution of EI across clusters. On the basis of both internal criteria and external evaluation, 3 clusters were selected as the optimal solution (Supplementary Table 3).

#### Descriptive statistics

Descriptive statistics for sociodemographic, socioeconomic factors, eating patterns, BMI categories, and diet quality (DGI score and its components) were reported as percentages or means (SD). The adjusted Pearson Chi-square test was used to evaluate the differences in categorical variables across different latent classes and clusters. Fisher’s exact test was applied to compare categorical variables when the expected frequency in any category was <5. The *F*-test was performed to assess the differences in continuous variables across latent classes and clusters, and the Bonferroni correction was applied to adjust for multiple comparisons across >2 patterns.

#### Comparison of LCA and cluster analysis

The 2 methods were compared using multiple complementary approaches. First, the derived TEPs were compared visually using pattern plots, which shows the peaks in timing of conditional probabilities of EOs occurrence in LCA and peaks in EI in MDTW-based clustering. Second, participant membership overlaps between latent classes and clusters of TEPs were used to assess similarity in group assignments. Third, kappa statistic was used to assess the agreement between the 2 classification methods.

Fourth, the strength of associations between TEPs derived from each method, overall diet quality, and BMI were compared, with higher *R*^2^ indicating stronger associations between TEPs, overall diet quality, and BMI. To check the assumptions for regression models, continuous variables were evaluated for normality using skewness, kurtosis, and the Shapiro–Wilk test. BMI was positively skewed (skewness = 2.20, kurtosis = 6.85) and deviated significantly from normality (Shapiro–Wilk W = 0.811, *P* < 0.001). To address non-normality, BMI was natural log-transformed. Adjusted geometric means of BMI were estimated by back-transforming predicted values from the log scale, with SEs calculated using the delta method. Pairwise group differences (β-coefficient ± SE) were derived from linear contrasts on the log scale and expressed as percent differences, with 95% confidence intervals (CIs) and *P* values obtained from multiple linear regression models. A linear regression model was also used to examine associations of classes and clusters of TEPs with overall diet quality. Models were adjusted for age, sex, education level, country of birth, household income (equivalized), smoking status, alcohol drinking, daily sleep duration, total EI, energy misreporting, and BMI (for diet quality models only) or DGI score (for BMI models only). Model fit and explanatory power were evaluated using adjusted *R*^2^. Multicollinearity among exposures and covariates was assessed using variance inflation factors, which ranged from 1 to 3.6, indicating no evidence of problematic multicollinearity. Model assumptions were further checked using residuals compared with fitted values plots. Results are reported as mean ± SE for DGI scores or geometric mean ± SE for BMI, with β coefficients (SE), 95% CIs, and adjusted *R*^2^ values.

Finally, the resultant TEPs were evaluated in relation to their ability to discriminate participants with high diet quality and obesity using the area under the receiver operating curve (ROC). AUC was calculated using predicted probabilities from logistic regression models examining the association of classes and clusters of TEPs with binary variables for diet quality (top tertile or bottom 2 tertiles) and BMI (persons with obesity or without obesity). The method with the higher AUC was considered potentially more suitable for deriving TEPs because it indicates a stronger ability to classify individuals as having high or low diet quality, and with or without obesity, with greater sensitivity and specificity. This reflects a more accurate detection of differences in TEPs associated with these outcomes. Differences in AUC were tested for statistical significance. A significance level of *P* < 0.05 was applied to all analyses. Statistical analyses were performed using Stata (version 18, Stata Corp).

## Results

### Characteristics of TEPs derived from LCA

**FIGURE 2:**
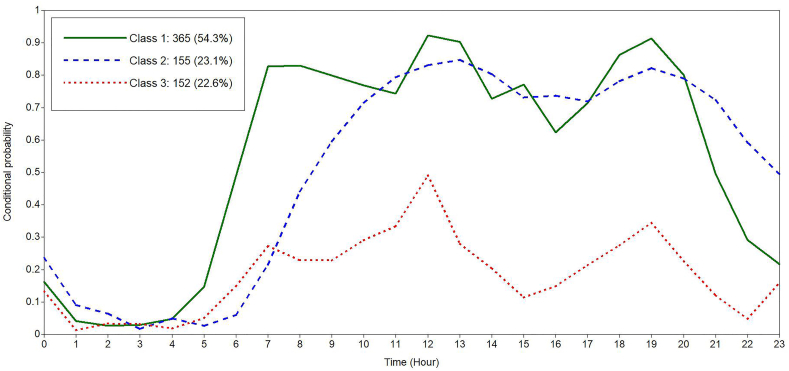
Conditional probabilities of eating occasion consumption at each hour of the day for the 3 classes of TEPs identified through latent class analysis among Australian adults (*n* = 672). TEP, temporal eating pattern.

The conditional probability of consuming EO across each hour of the day for the 3 latent classes of TEPs is illustrated in Figure 2. Class 1 of TEPs (*n* = 365, 54.3%) showed higher conditional probabilities (>0.8) of consuming EOs during 07:00–08:00, 12:00, and 19:00 h, reflecting conventional mealtimes in the Australian context. Class 2 of TEPs (*n* = 155, 23.1%) had a higher conditional probability of consuming EO at 13:00 and 19:00 h, with an additional higher conditional probability of eating after 21:00 h compared with the other classes (i.e., a later eating pattern). Class 3 of TEPs (*n* = 152, 22.6%) showed modest probabilities of consuming EOs throughout the day, with no conditional probability peak exceeding 0.5.

### Characteristics of TEPs derived from MDTW-based cluster analysis

**FIGURE 3:**
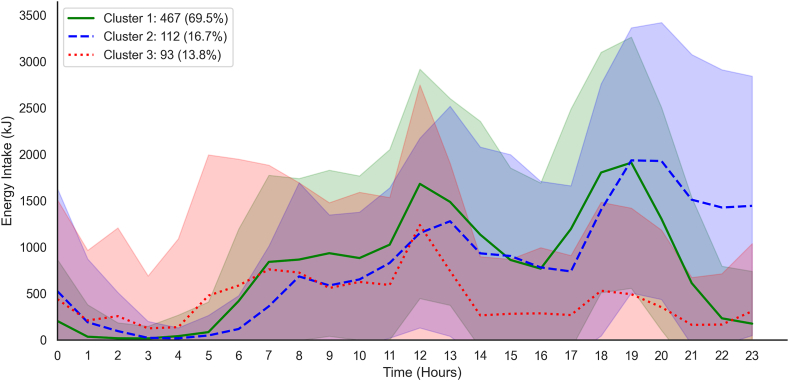
Mean hourly energy intake including ± SD throughout the day across 3 clusters of TEPs identified using MDTW-based cluster analysis among Australian adults (*n* = 672). MDTW, modified dynamic time warping; TEP, temporal eating pattern.

The distribution of EI at each hour of the day for the 3 clusters of TEPs identified using MDTW-based cluster analysis is displayed in Figure 3. Cluster 1 of TEPs (*n* = 467, 69.5%) showed distinct peaks of EI during 07:00–9:00, 12:00, and 18:00–19:00 h, corresponding to breakfast, lunch, and dinner mealtimes in the Australian context, respectively. Cluster 2 of TEPs (*n* = 112, 16.7%) showed EI peaks at 8:00, 13:00, and between 19:00 and 21:00 h, indicating a delayed lunch and greater energy consumption in the evening compared with the other 2 clusters. Cluster 3 of TEPs (*n* = 93, 13.8%) had lower EI throughout the day compared with the other 2 clusters, with modest peaks at 07:00 and 12:00 h, and a small peak between 18:00 and 19:00 h that indicates groups with moderate EI concentrated earlier in the day.

**FIGURE 4:**
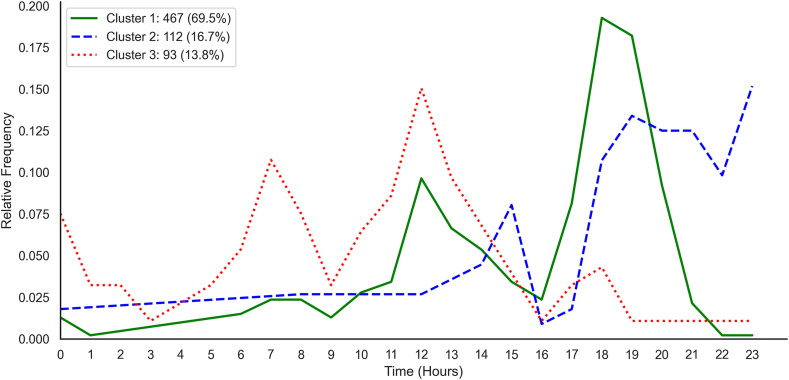
Time of the highest energy intake throughout the day across clusters of TEPs identified using MDTW-based cluster analysis among Australian adults (*n* = 672). MDTW, modified dynamic time warping; TEP, temporal eating pattern.

The relative frequency of the largest EI event of clusters of TEPs is displayed in Figure 4. Cluster 1 of TEPs had the largest EI at 12:00 and 18:00–19:00 h. In contrast, Cluster 2 of TEPs tended to consume their largest EI predominantly after 20:00 h. Cluster 3 of TEPs had the largest EI at 07:00 and 12:00 h.

### Agreement between TEPs derived from LCA compared with MDTW-based cluster analysis

The highest percentage of participant membership overlap occurred between Class 3 and Cluster 3 of TEPs (73.1%), and the least between Class 2 and Cluster 3 of TEPs (3.2%). A moderate degree of participant membership overlap was observed between the classes and clusters of TEPs. Specifically, 66.8% of participants in Class 1 were also in Cluster 1 of TEPs, 56.2% of participants in Class 2 were in Cluster 2 of TEPs, and 73.1% of participants in Class 3 were in Cluster 3 of TEPs (Supplementary Table 4). The kappa statistic (0.38) indicated fair overall agreement between TEPs derived by LCA and MDTW-based cluster analysis [50]. Both methods assigned participants to the same group ∼65.9% of the time, which substantially exceeded the 44.7% expected by chance. This agreement was statistically significant (*P* < 0.001).

### Sociodemographic profiles across TEPs derived from LCA compared with MDTW-based cluster analysis

The characteristics of participants across classes and clusters of TEPs are presented in Table 1. Class 1 and Cluster 1 (“Conventional” TEP) had the largest proportion of participants compared with the other 2 classes and clusters. Age, relationship status, and energy misreporting were statistically significant differences across both classes and clusters of TEPs (all *P* < 0.05). Among classes of TEPs, significant differences were also observed for education level, occupation, shift work, and work hours. In contrast, sex distribution differed significantly only across clusters of TEPs. No significant differences were observed by country of birth, household income, smoking status, alcohol use, physical activity, or sleep duration among both groups of TEPs. Overall, participants in Class 1 and Cluster 1 of TEPs were older, married, and more likely to report plausible EI. Class 1 of TEPs also had higher proportions of tertiary-educated adults, professional workers, and nonshift workers, whereas adults in Class 3 had longer work hours. Cluster 1 included a higher proportion of females, whereas Cluster 3 had more males compared with the other 2 clusters. The higher proportion of energy under-reporters was represented in both Class 3 (59.2%) and Cluster 3 (58.1%) of TEPs.

**TABLE 1:** Characteristics of participants across classes and clusters of TEPs derived by latent class analysis and MDTW-based cluster analysis among Australian adults aged 18–65 y (*n* = 672).

Characteristics	Total (*n* = 672)	Latent class analysis				Cluster analysis			
		Class 1(*n* = 365)	Class 2 (*n =* 155)	Class 3 (*n =* 152)	*P* value	Cluster 1 (*n =* 467)	Cluster 2 (*n =* 112)	Cluster 3 (*n =* 93)	*P* value
Age^1^ (y)	35.4 (34.4, 36.5)	37.7 (36.3, 39.1)^a^	33.1 (31.0, 35.1)^b^	32.4 (30.4, 34.3)^b^	<0.001	36.3 (35.1, 37.6)^a^	33.3 (31.0, 35.6)^b^	33.5 (31.0, 36.1)^b^	0.032
Sex (%)									
Male	15.6	13.4	16.1	20.4	0.136	13.1^a^	17.9	25.8^b^	0.007
Female	84.4	86.6	83.9	79.6		86.9	82.1	74.2	
Education qualification (%)									
Grade ≤12	8.5	6.8	8.4	12.5	<0.001	8.8	7.1	8.6	0.623
Still studying for the first qualification	15.3	9.9^a^	23.9^b^	19.7		13.5	20.5	18.3	
Certificate	28.3	29.6	24.5	28.9		28.9	25.9	28.0	
Tertiary	47.9	53.7	43.2	38.8		48.8	46.4	45.2	
Country of birth (%)									
Australia	74.9	75.9	71.6	75.7	0.570	76.7	68.8	73.1	0.204
Outside Australia	25.1	24.1	28.4	24.3		23.3	31.2	26.9	
Relationship status (%)									
Married/living together	44.3	54.0^a^	32.3^b^	33.6^b^	<0.001	59.9^a^	28.6^b^	35.5	<0.001
In a relationship but not living together	19.1	16.2	24.5	20.4		17.3	26.8	18.3	
Not in a relationship	36.6	29.9^a^	43.2^b^	46.0^b^		32.8	44.6	46.2	
Main occupation (%)									
Manager/professional	30.1	34.8^a^	20.6^b^	28.3^a^	0.033	29.3	27.7	36.6	0.542
Technician/trade worker/machinery operator/driver/laborer	2.2	2.7	1.3	2.0		1.9	1.8	4.3	
Community worker/personal service worker/clerical worker/administrative worker/sales worker	15.8	15.3	16.1	16.4		16.1	17.0	12.9	
No paid job/student/other	51.9	47.1	61.9	53.3		52.7	53.6	46.2	
Equivalized household income level (AUD/wk) (%)									
Quartile 1 (0–900)	21.0	19.5	27.7	17.8	0.060	20.6	25.9	17.2	0.134
Quartile 2 (901–1429)	18.8	19.7	15.5	20.4		17.3	19.6	25.8	
Quartile 3 (1430–2250)	20.4	22.5	14.3	21.7		22.3	11.6	21.5	
Quartile 4 (>2251)	18.8	21.3	13.5	17.8		18.2	19.6	20.4	
Did not answer	21.0	17.0	29.0	22.3		21.6	23.3	15.1	
Shift work (*n* = 346) (%)									
Yes	13.1	9.9^a^	16.8^b^	17.1^b^	0.025	12.6	11.6	17.2	0.431
No	86.9	90.1^a^	83.2^b^	82.9^b^		87.4	88.6	82.8	
Work hours per week (*n* = 346)	34.7 (33.0, 36.4)	33.7 (31.6, 35.8)^a^	31.9 (28.2, 35.6)^a^	40.0 (36.1, 43.8)^b^	0.004	34.5 (32.6, 36.5)	33.6 (29.3, 37.8)	36.8 (31.9, 41.8)	0.793
Smoking status^2^ (%)									
Nonsmoker	72.5	73.4	72.3	70.4	0.779	72.2	75.9	69.9	0.610
Past or current smoker	27.5	26.6	27.7	29.6		27.8	24.1	30.1	
Alcohol consumption of ≤4 standard drinks per day (%)									
Yes	20.8	21.1	22.6	18.4	0.658	21.6	19.6	18.3	0.725
No	79.2	78.9	77.4	81.6		78.4	80.4	81.7	
Met physical activity guidelines^3^ (%)									
Yes	76.9	77.5	76.1	76.3	0.922	76.9	77.7	76.3	0.973
No	23.1	22.5	23.9	23.9		23.1	22.3	23.7	
Daily weekday sleep time^1^ (h)	7.3 (7.2, 7.4)	7.3 (7.2, 7.4)	7.4 (7.1, 7.7)	7.2 (7.0, 7.4)	0.469	7.3 (7.2, 7.4)	7.2 (7.0, 7.5)	7.5 (7.2, 7.8)	0.123
Energy misreporting status^4^ (%)									
Plausible reporters	70.5	83.0^a^	71.0^a^	40.1^b^	<0.001	76.0^a^	71.4^a^	41.9^b^	<0.001
Under-reporter	28.4	15.6^a^	28.4^a^	59.2^b^		22.5^a^	28.6^a^	58.1^b^	
Over-reporter	1.1	1.4	0.6	0.7		1.5	0.0	0.0	
BMI category^5^^(^%)									
Healthy weight	47.6	47.1	50.4	46.1	0.311	46.0	48.2	54.8	0.462
Overweight	28.4	30.7	21.9	29.6		30.2	25.0	23.7	
Obesity	24.0	22.2	27.7	24.3		23.8	26.8	21.5	

Differences between classes and clusters of TEPs for continuous variables were assessed by using ANOVA, with superscripts showing pairwise differences compared using *F*-tests adjusted using the Bonferroni correction. Differences in categorical variables were assessed using Pearson’s chi-square test, except for “Main occupation” and “Energy misreporting status,” for which Fisher’s exact test was used.

Abbreviations: ANOVA, analysis of variance; CI, confidence interval; MDTW, modified dynamic time warping; TEP, temporal eating pattern.

1 Values are means (95% CIs) or percentages unless otherwise stated.

2 Shift workers (*n* = 38): rotating shifts without night (*n* = 12); rotating shifts with night (*n* = 20); permanent night shift (*n* = 2); other (e.g., working from home as required) (*n* = 4).

3 Whether met the Physical Activity Guidelines of 150 min and 5 sessions/wk.

4 Defined by using the ±SD cutoff for the ratio of energy intake to energy expenditure of <0.44 for under-reporters and >1.56 for over-reporters.

5 Defined as normal weight for BMI (kg/m^2^) <25, overweight for BMI: 25.0–29.9, obesity for BMI ≥30.0. Within the healthy weight category, a small proportion of participants were underweight: 1% in Class 1, 1% in Class 2, and 3% in Class, and 1% in Cluster 1, 4% in Cluster 2, and 2% in Cluster 3.

### Eating pattern characteristics across TEPs derived from LCA compared with MDTW-based cluster analysis

Differences in eating pattern characteristics across classes and clusters of TEPs are presented in Table 2. Frequencies of EOs, meals, snacks, and total daily EI varied significantly across all TEPs (all *P* < 0.001). The highest frequency of EO, meal, and snack was observed in Class 1 and Cluster 1 of TEPs, whereas the lowest was observed in Class 3 and Cluster 3 of TEPs compared with the other classes and clusters. The proportion of EI from meals and snacks differed significantly only across classes of TEP, with Class 3 showing a higher proportion of EI from meals (81.9%; *P* < 0.001) compared with the other 2 classes, and Class 2 showed a higher proportion from snacks (27.1%; *P* < 0.001). The lowest proportion of EI from snacks was observed among Class 3 of TEPs (18.1%).

**TABLE 2:** Eating pattern characteristics of classes and clusters of TEPs derived by latent class analysis and MDTW-based cluster analysis^1^, respectively (*n* = 672).

Characteristics	Latent class analysis				Cluster analysis			
	Class 1	Class 2	Class 3	*P* value	Cluster 1	Cluster 2	Cluster 3	*P* value
EO frequency	4.4 (4.3, 4.5)^a^	3.6 (3.4, 3.8)^b^	2.1 (2.0, 2.3)^c^	<0.001	4.0 (3.9, 4.1)^a^	3.5 (3.2, 3.8)^b^	2.4 (2.1, 2.7)^c^	<0.001
Meal frequency	2.9 (2.8, 2.9)^a^	2.4 (2.3, 2.5)^b^	1.8 (1.6, 1.9)^c^	<0.001	2.7 (2.6, 2.7)^a^	2.4 (2.2, 2.5)^b^	1.8 (1.6, 2.0)^c^	<0.001
Snack frequency	1.8 (1.8, 1.9)^a^	1.6 (1.5, 1.7)^b^	0.7 (0.6, 0.9)^c^	<0.001	1.7 (1.6, 1.7)^a^	1.5 (1.4, 1.7)^a^	0.9 (0.8, 1.1)^c^	<0.001
Energy intake (MJ)	7.1 (6.9, 7.3)^a^	6.4 (6.1, 6.7)^b^	4.1 (3.7, 4.5)^c^	<0.001	6.6 (6.4, 6.8)^a^	6.1 (5.7, 6.6)^a^	4.4 (3.8, 5.0)^c^	<0.001
Total EI from meals (%)	76.5 (75.4, 77.6)^a^	72.9 (71.0, 74.8)^b^	81.9 (77.7, 86.1)^c^	<0.001	77.1 (76.0, 78.3)	75.0 (72.5, 77.5)	77.5 (71.6, 83.4)	0.215
Total EI from snacks (%)	23.5 (22.4, 24.6)^a^	27.1 (25.2, 29.0)^b^	18.1 (13.9, 22.3)^c^	<0.001	22.9 (21.7, 24.0)	25.0 (22.5, 27.5)	22.5 (16.6, 28.4)	0.215

Significant differences between classes and clusters of TEPs were assessed using ANOVA (*P* <0.05), with superscripts showing pairwise differences compared using *F*-tests adjusted using the Bonferroni correction.

Abbreviations: ANOVA, analysis of variance; CI, confidence interval; EI, energy intake; EO, eating occasion; MDTW, modified dynamic time warping; TEP, temporal eating pattern.

1 Values are means (95% CIs) or percentages unless otherwise stated.

### Diet quality across TEPs derived from LCA compared with MDTW-based cluster analysis

Differences in DGI scores and intakes of food groups (DGI components) across classes and clusters of TEPs are shown in Table 3. Statistically significant differences in DGI scores, fluid, and discretionary food consumption were observed across both TEP classes and clusters. Vegetable and fruits intakes were statistically significantly different between classes and clusters, respectively, whereas grains, lean meat/alternatives, and dairy/alternatives showed no statistically significant differences. Class 1 had the highest mean DGI score 70.3 (95% CI: 69.1, 71.6; *P* < 0.001), vegetable serve 4.1 (95% CI: 3.9, 4.4; *P* = 0.010), and fluid serve 6.0 (95% CI: 5.6, 6.4; *P* < 0.001) compared with the other 2 classes. Among clusters of TEPs, both Cluster 1 and Cluster 3 had higher mean DGI scores 68.7 (95% CI: 67.6, 69.8), and 68.8 (95% CI: 66.0, 71.6), respectively, compared with Cluster 2 (65.7; 95% CI: 63.5, 67.9); *P* < 0.045. Cluster 1 had a higher fruit serve intake 0.9 (95% CI: 0.8, 1.0; *P* = 0.018) compared with the other 2 clusters. In addition, discretionary food intake was higher in cluster 1 (3.7; 95% CI: 3.5, 3.9) and cluster 2 (3.8; 95% CI: 3.4, 4.3) compared with cluster 3 (2.0; 95% CI: 1.6, 2.4); *P* = 0.013.

**TABLE 3:** Diet quality scores and servings of food group consumption by TEPs by latent class analysis and MDTW-based cluster analysis among Australian adults^1^, (*n* = 672).

Characteristics	Latent class analysis				Cluster analysis			
	Class 1	Class 2	Class 3	*P* value	Cluster 1	Cluster 2	Cluster 3	*P* value
Dietary Guidelines Index (range: 0–120)^2^	70.3 (69.1, 71.6)^a^	64.8 (63.1, 66.6)^b^	66.7 (64.8, 68.6)^c^	<0.001	68.7 (67.6, 69.8)^a^	65.7 (63.5, 67.9)^b^	68.8 (66.0, 71.6)^a^	0.045
Serves of food group (recommended number of daily serves)^3^								
Vegetables (5–6)	4.1 (3.9, 4.4)^a^	3.2 (2.9, 3.4)^b^	2.2 (1.9, 2.6)^c^	0.010	3.8 (3.6, 4.0)	3.2 (2.7, 3.7)	2.3 (1.9, 2.7)	0.282
Fruits (2–2)^4^	1.0 (0.9, 1.1)	0.7 (0.6, 0.9)	0.5 (0.4, 0.7)	0.194	0.9 (0.8, 1.0)^a^	0.8 (0.6, 1.0)^b^	0.7 (0.4, 1.0)^c^	0.018
Grain foods (4–6)	4.5 (4.3, 4.7)	4.2 (3.9, 4.5)	2.8 (2.5, 3.1)	0.511	4.3 (4.1, 4.5)	4.0 (3.7, 4.3)	3.0 (2.5, 3.4)	0.408
Lean meats/alternatives (2–3)	2.2 (2.1, 2.3)	1.9 (1.7, 2.0)	1.3 (1.1, 1.5)	0.603	2.0 (1.9, 2.1)	1.8 (1.6, 2.1)	1.3 (1.1, 1.5)	0.103
Dairy/alternatives (2–4)	1.5 (1.4, 1.6)	1.2 (1.1, 1.3)	0.8 (0.7, 0.9)	0.276	1.3 (1.3, 1.4)	1.1 (1.0, 1.2)	1.0 (0.8, 1.2)	0.075
Fluids^5^	6.0 (5.6, 6.4)^a^	4.3 (3.8, 4.8)^b^	2.5 (2.1, 2.9)^b^	<0.001	5.3 (5.0, 5.7)^a^	4.0 (3.4, 4.6)^b^	3.3 (2.6, 4.0)^b^	0.041
Discretionary foods^6^	3.8 (3.6, 4.0)^a^	4.0 (3.6, 4.3)^a^	2.1 (1.7, 2.4)^b^	0.002	3.7 (3.5, 3.9)^a^	3.8 (3.4, 4.3)^a^	2.0 (1.6, 2.4)^b^	0.013

Different superscript letters indicate significant *F*-test comparisons, with adjustment for multiple comparisons, between Classes and Clusters (significance level: adjusted *P* < 0.05).

Abbreviations: CI, confidence interval; MDTW, modified dynamic time warping; TEP, temporal eating pattern.

1 Values are mean estimates (95% CIs) adjusted for age (y), sex, education qualification, country of birth, equivalized household income, smoking status, alcohol consumption, daily sleep time, total daily energy intake, energy misreporting, and BMI.

2 The Dietary Guidelines Index represents a total diet quality score with higher scores indicating better overall diet quality.

3 Vegetables, fruits, grains, lean meats/alternatives, and dairy/alternatives are the 5 food groups recommended for adequate intake, whereas discretionary foods are recommended to be consumed only sometimes and in small amounts.

4 Exclude fruit juices.

5 Fluids include water and all types of beverages.

6 Include fruit juices, and 1 serving = 600 kJ.

### Associations of classes and clusters of TEPs with overall diet quality and BMI

The associations of classes and clusters of TEPs with overall diet quality are shown in Table 4. Both classes and clusters of TEPs were significantly associated with overall diet quality. The strongest association among TEP classes was observed between Class 1 and Class 2, with Class 1 showing significantly higher mean DGI scores (β = 4.8 ± 1.2, *P* < 0.001). Similarly, a modestly significant association was found between TEP clusters, with participants in Cluster 1 having a higher diet quality score compared with those in Cluster 2 (β = 2.6 ± 1.3, *P* = 0.045). Classes and clusters of TEPs explained 6% and 4% of the variance in DGI scores, respectively. Although no statistically significant associations were observed between classes or clusters of TEPs and BMI, both classification approaches explained ∼13% of the variance in geometric mean BMI each, as indicated by the adjusted *R*^2^ (Table 5).

**TABLE 4:** Adjusted regression model estimates of mean DGI score across classes and clusters of TEPs identified by latent class analysis and MDTW-based cluster analysis, respectively, among Australian adults (n = 672).

Latent class analysis								
Adjusted models^1^	DGI score (SE)^2^	β^3^ ± SE Compared to Class 2^3^	95% CI	*P*-value	β^3^ ± SE Compared to Class 3^3^	95% CI	*P*-value	Adjusted R^2^
Class 1	70.3 (0.6)	4.8 ± 1.2	2.5, 7.1	<0.001	3.0 ± 1.3	0.3, 5.6	0.027	0.06
Class 2	64.8 (0.9)				–1.8 ± 1.4	–4.6, 1.1	0.219	
Class 3	66.7 (1.0)							

Cluster analysis								
Adjusted models^1^	DGI score (SE)^2^	β^3^ ± SE Compared to Cluster 2^3^	95% CI	*P*-value	β^3^ ± SE Compared to Cluster 3^3^	95% CI	*P*-value	Adjusted R^2^
Cluster 1	68.7 (0.6)	2.6 ± 1.3	0.1, 5.1	0.045	–0.2 ± 1.5	–3.1, 2.6	0.885	0.04
Cluster 2	65.7 (1.1)				–2.8 ± 1.7	–6.2, 0.6	0.111	
Cluster 3	68.8 (1.4)							

Abbreviations: DGI, Dietary Guideline Index; SE, standard error.

Significance level: adjusted P< 0.05.

1 Models were adjusted for age (years), sex, education qualification, country of birth, equivalised household income, smoking status, alcohol consumption, daily sleep time, total daily energy intake, energy misreporting and BMI.

2 Values are mean and standard error of the mean.

3 ß represents the difference in mean DGI between two compared classes or clusters of TEPs.

**TABLE 5:** Adjusted regression model estimate of geometric mean BMI (kg/m^2^) across classes and clusters of TEPs identified by latent class analysis and MDTW-based cluster analysis, respectively, in Australian adults (n = 672).

Latent class analysis								
Adjusted Models^1^	BMI (SE) 2	β^2^±SE Compared to Class 2^3^	95% CI	*P*-value	β^2^±SE Compared to Class 3^3^	95% CI	*P*-value	Adjusted R^2^
Class 1	25.1 (0.4)	–3.3 ± 2.2	–7.4, 1.0	0.133	–2.5 ± 2.6	–7.3, 2.4	0.312	0.13
Class 2	26.0 (0.6)				0.8 ± 2.8	–4.5, 6.3	0.774	
Class 3	25.8 (0.7)							

Cluster analysis								
Adjusted models^1^	BMI (SE) 2	β^2^±SE Compared to Cluster 2^3^	95% CI	*P*-value	β^2^±SE Compared to Cluster 3^3^	95% CI	*P*-value	Adjusted R^2^
Cluster 1	25.3 (0.4)	–2.7 ± 2.4	–7.1, 1.9	0.251	2.3 ± 2.8	–3.0, 8.0	0.394	0.13
Cluster 2	26.0 (0.7)				5.2 ± 3.3	–1.3, 12.1	0.119	
Cluster 3	24.8 (0.7)							

Abbreviations: BMI (kg/m^2^), body mass index; SE, standard error.

Akaike information criterion (AIC) model fit for LCA derived TEPs was 248.3 and 248.1 for MDTW-based clustering.

Significance level: adjusted P< 0.05.

1 Models were adjusted for age (years), sex, education qualification, country of birth, equivalised household income, smoking status, alcohol consumption, daily sleep time, total daily energy intake, energy misreporting and DGI scores.

2 Values are adjusted geometric mean and standard error.

3 β represents the adjusted difference in log-transformed BMI between groups. Values are reported as back-transformed percent differences relative to the reference group.

### Discriminating adults with higher diet quality and obesity using TEPs derived from LCA compared with MDTW-based cluster analysis

**FIGURE 5:**
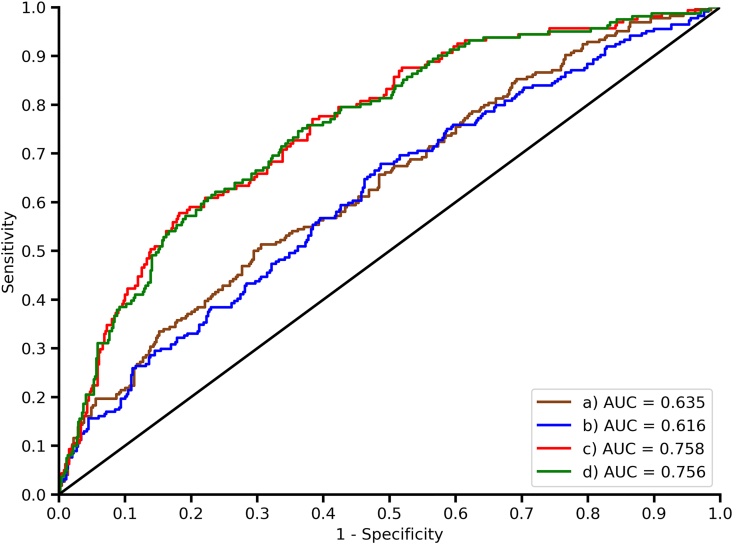
Area under the receiver operating curve using predicted probabilities from multiple logistic regression models of (A) latent classes and (B) clusters in relation to high diet quality, and (C) latent classes, and (D) clusters in relation to obesity (BMI ≥30).

The AUC was used to examine how well TEPs identified from LCA and MDTW-based cluster analysis could discriminate participants with higher diet quality or obesity. Predicted probabilities from logistic regression models for both methods were used to conduct ROC analysis. For discriminating a higher diet quality, the AUC was 0.635 for classes and 0.616 for clusters of TEPs, with no significant difference between methods (*P* = 0.565). For obesity discrimination, AUC values were approximately similar between methods (0.758 compared with 0.756, respectively; *P* = 0.934) (Figure 5).

## Discussion

Our study compared 2 common methods for identifying TEPs (LCA and MDTW-based clustering) and examined associations with diet quality and obesity. LCA classified individuals based on the presence/absence of EOs at each hour, whereas MDTW-based clustering used continuous hourly EI. Both methods identified 3 distinct and visually comparable TEPs with moderate membership overlap and fair agreement. Additionally, both methods identified TEPs that explained comparable variance in BMI (∼13%) with minimal differences in diet quality prediction (LCA: 6% vs MDTW: 4%). However, MDTW-based clustering identified more interpretable TEPs by integrating continuous hourly energy intake amounts compared with latent profile analysis (LPA) (continuous form of LCA). This methodological advantage allows MDTW-based clustering to characterize individuals following specific patterns as light or heavy eaters at specific times, eating behavioral nuances that LCA's binary classification misses.

To our knowledge, this is the first study comparing methods that integrate timing and dietary data to identify TEPs. Both LCA and MDTW-based clustering identified 3 distinct TEPs with comparable peak timing. The first patterns (Class 1/Cluster 1) reflected conventional patterns with evenly spaced EOs or 3 main energy peaks [9,[11], [12], [13],^15^,^18^]. The second patterns (Class 2/Cluster 2) showed later EOs or EI, whereas the third (Class 3/Cluster 3) showed earlier-day concentrated intake. Small differences in later-day peaks (19:00 h compared with 18:00 h) among Class 3 and Cluster 3 indicate that energy peaks do not always coincide with peaks in EOs, a nuance captured by MDTW-based clustering but potentially missed by LCA. This likely reflects that MDTW accounts for both timing and intake amount, whereas LCA considers only the presence of EOs.

Our study found that Class 2 was associated with higher snack-derived EI, likely reflecting the energy dense nature of snack foods. Evidence suggests that both snack timing and quality influence metabolic outcomes, where earlier nutrient-dense intake is beneficial and late-night or irregular eating is detrimental [[51], [52], [53], [54], [55]]. In contrast, Class 3 and Cluster 3 showed fewer EOs and lower EI, likely due to under-reporting or genuinely lower intake, highlighting the utility of TEPs methods in identifying under-reporters and the need for objective dietary assessment tools [56]. Additionally, high discretionary food intake was observed not only in later eating patterns but also in conventional patterns, which differs from previously studies, potentially due to intrapersonal-, social- and environmental-contextual factors related to eating [[57], [58], [59], [60], [61]]. The patterns with earlier energy concentration showed lower discretionary food intake, consistent with evidence that morning-focused eating may reduce unhealthy food intake overall [62,63].

Our study found that TEPs identified using LCA explained marginally more variation in diet quality than those from MDTW-based clustering. Patterns with evenly spaced or earlier EOs, such as Class 1 and Cluster 3, compared with patterns with later intake, consistent with previous studies [13,22,64,65]. These findings highlight that the timing and distribution of EOs/EI influence dietary quality. Promoting evenly spaced meals or earlier EI may improve diet quality at the population level, and public health strategies needs to encourage structured meal timing and reduced late day EI to lower the risk of obesity and other diet-related chronic conditions.

This study found no association between TEPs and obesity, although LCA and MDTW clustering explained similar variance in BMI. Previous studies reported mixed findings, with some linking grazing or later energy peaks to higher obesity risk [15,22] and others showing no association or lower risk in patterns with evenly distributed EI [11,18]. These inconsistencies may be attributed to methodological heterogeneity or population differences across studies. The null association in our study may be due to limited BMI variation, largely healthy participants, and the cross-sectional design. Nonetheless, both methods may still capture variation in other health outcomes [10,66]. Future studies using probability sampling, longitudinal or interventional designs, and alternative adiposity measures are needed to assess causality.

Our findings indicate that sociodemographic differences were observed across TEPs, with older and married adults were more often represented in TEPs with conventional type patterns, consistent with previous studies [9,10,13,15,18]. In contrast, TEP with later energy peaks (Class 2) were more common among unemployed individuals and students, who are prone to skipping breakfast, delayed eating, and later snacking, potentially influenced by food insecurity, or disrupted routines [[67], [68], [69], [70]]. However, direct evidence on the association between TEPs and food insecurity remains limited. Additionally, sociodemographic differences were more pronounced across LCA-derived TEPs than MDTW-based clustering, which likely reflects participant characteristics rather than methodological differences.

Methods used to derive dietary patterns often yield overlapping yet distinct patterns, which influence both participant classification and observed associations [[71], [72], [73]]. In this study, LCA and MDTW-based clustering identified broadly similar TEPs but differed in group membership and sensitivity to outcome variability, likely due to differences in input data [71,74]. Notably, LPA, a form of LCA suitable for continuous intake data, failed to yield meaningful patterns (Supplementary Figure 1), whereas MDTW-based clustering was able to successfully capture the timing, quantity, and sequence of intake [14]. Thus, the MDTW-based clustering method may offer flexibility to integrate multidimensional lifestyle data (e.g., diet, physical activity, and sleep) [75], to understand their combined health effects in future research.

This study’s strengths include the direct comparison of LCA and MDTW-based clustering for deriving TEPs using fine-grained hourly EOs and EI data, which may advance chrononutrition research beyond traditional broad time blocks (such as morning, midday, or evening), with comparable predictive performance supporting robustness while highlighting method-specific influences on associations with diet quality and obesity. Additional strengths include the use of MDTW with a 3-h constraint to avoid implausible alignments and real-time multiday dietary recording via the “FoodNow” app to reduce recall bias. However, limitations include a small, potentially nonrepresentative sample, cross-sectional design limiting causal inference, weekday and weekend data heterogeneity, inability to control for shift work, reliance on self-reported measures prone to error and under-reporting, and modest cluster stability, which warrants cautious interpretation [76].

In our study, TEPs were associated with diet quality but not obesity, underscoring the need for future research using objective adiposity measures and interventional trials to establish causality. Such research could inform dietary recommendations on optimal timing and quantity of EI, while examining the joint effects of TEPs and diet quality may clarify their role in obesity risks. Additionally, examining temporal patterns of specific food groups could provide further insight into health impacts, and the lack of meaningful patterns from LPA suggests that alternative extensions of LCA should be considered in future research.

In conclusion, both LCA and MDTW-based clustering identified comparable TEPs, with patterns characterized by evenly spaced EOs or 3 energy peaks associated with favorable diet quality compared with patterns with later EOs or EI, and no associations with BMI. LCA explained slightly more variance in diet quality; however, both methods showed modest explanatory power. MDTW-based clustering identified more meaningful TEPs by integrating both the timing and continuous EI compared with LPA (continuous type of LCA). Overall, these findings highlight both methods are suitable for deriving TEPs, and the need for future studies using objective adiposity measures and experimental designs to clarify the health effects of TEPs.

## Author contributions

The authors’ responsibilities were as follows – SAM, DWD: were chief investigators of the Australian Research Council Discovery Project grant and led the “EveryDayLife” project design and implementation; BRJ, RML, DWD, SAM: designed the analysis for this study; BRJ: conducted the statistical analyses with support from AZK and RML; BRJ: also drafted the manuscript; and all authors: reviewed it critically and approved the final version for publication.

## Data availability

The datasets used in our study are not publicly available because of Deakin University’s Human Research Ethics Committee procedure, but they can be requested from the senior authors.

## Funding

“EveryDayLife” was supported by an Australian Research Council Discovery Grant [DP170100544]. BRJ is supported by the Deakin University Postgraduate Research Scholarship.

## Declaration of generative AI and AI-assisted technologies in the writing process

The author(s) declare that no generative AI or AI-assisted technologies were used in the writing of this manuscript.

## Conflict of interest

The authors report no conflicts of interest.
