# Supplementary material for: Identifying temporal eating patterns: a comparison of latent class analysis and dynamic time warping-based cluster analysis
Source: Am J Clin Nutr. 2026 Apr 15;123(6):101317. doi: 10.1016/j.ajcnut.2026.101317 (PMC13269345; doi:10.1016/j.ajcnut.2026.101317)
Supplement: Multimedia component 1 [file mmc1.docx]

Identifying temporal eating patterns: A comparison of latent class analysis and dynamic time warping-based cluster analysis: Beshada Rago Jima “Online Supplementary Material”

**Supplementary file 1, table 1:** Model fit indices for latent class models using latent class analysis

|  | **2 Classes** | **3 Classes** | **4 Classes** | **5 Classes** | **6 Classes** |
| --- | --- | --- | --- | --- | --- |
| AIC | 15807 | 15616 | 15579 | 15540 | 15510 |
| BIC | 16028 | 15950 | 16026 | 16099 | 16183 |
| Adjusted BIC | 15873 | 15715 | 15712 | 15706 | 15709 |
| LMR-ALRT | 1336, P<0.001 | 189, P =0.048 | 136, P =0.557 | 88, P=0.256 | 79, P=0.782 |
| BS-LRT | -8527, P<0.001 | -7829, P<0.001 | -7759, P<0.001 | -7690, P<0.001 | -7646, P<0.001 |

AIC: Akaike Information Criterion, BIC: Bayesian Information Criterion, BS: Bootstrap, LMR: Lo-Mendell Rubin, ALRT: Adjusted likelihood ratio test

**Supplementary file 1, table 2:** Mean latent class probabilities for most likely latent class membership of 3 latent classes^1^.

| Most likely class | Class 1 | Class 2 | Class 3 |
| --- | --- | --- | --- |
| Class 1 | 0.843 (0.143) | 0.137 (0.136) | 0.019 (0.066) |
| Class 2 | 0.095 (0.133) | 0.896 (0.138) | 0.008 (0.039) |
| Class 3 | 0.018 (0.050) | 0.023 (0.074) | 0.959 (0.096) |

^1^Values are mean and standard deviation.

**Supplementary file 1, table 3:** Silhouette and Dunn index values ^1^ of internal cluster variance and consistency for two to six clusters partitioning Australian adults (n, 672).

| Cluster K Partitions | | | | | |
| --- | --- | --- | --- | --- | --- |
|  | **K2** | **K3** | **K4** | **K5** | **K6** |
| Silhouette Index | 0.059 | 0.073 | 0.045 | 0.040 | 0.042 |
| Dunn Index | 0.226 | 0.221 | 0.244 | 0.244 | 0.226 |

^1^higher values indicate better clustering where energy intakes are similar within a cluster and more dissimilar between clusters.

**Supplementary file 1, table 4:** Participants’ membership overlaps between TEPs identified by latent class analysis and MDTW-based cluster analysis among Australian adults^1^, respectively (n = 672).

| **Latent class analysis** | **Cluster analysis** | | |  |
| --- | --- | --- | --- | --- |
|  | Cluster 1 | Cluster 2 | Cluster 3 |  |
| Class 1 | 312 (66.8) | 31 (27.7) | 22 (23.7) | |
| Class 2 | 89 (19.1) | 63 (56.2) | 3 (3.2) | |
| Class 3 | 66 (14.1) | 18 (16.1) | 68 (73.1) | |

^1^ Number of samples (%)


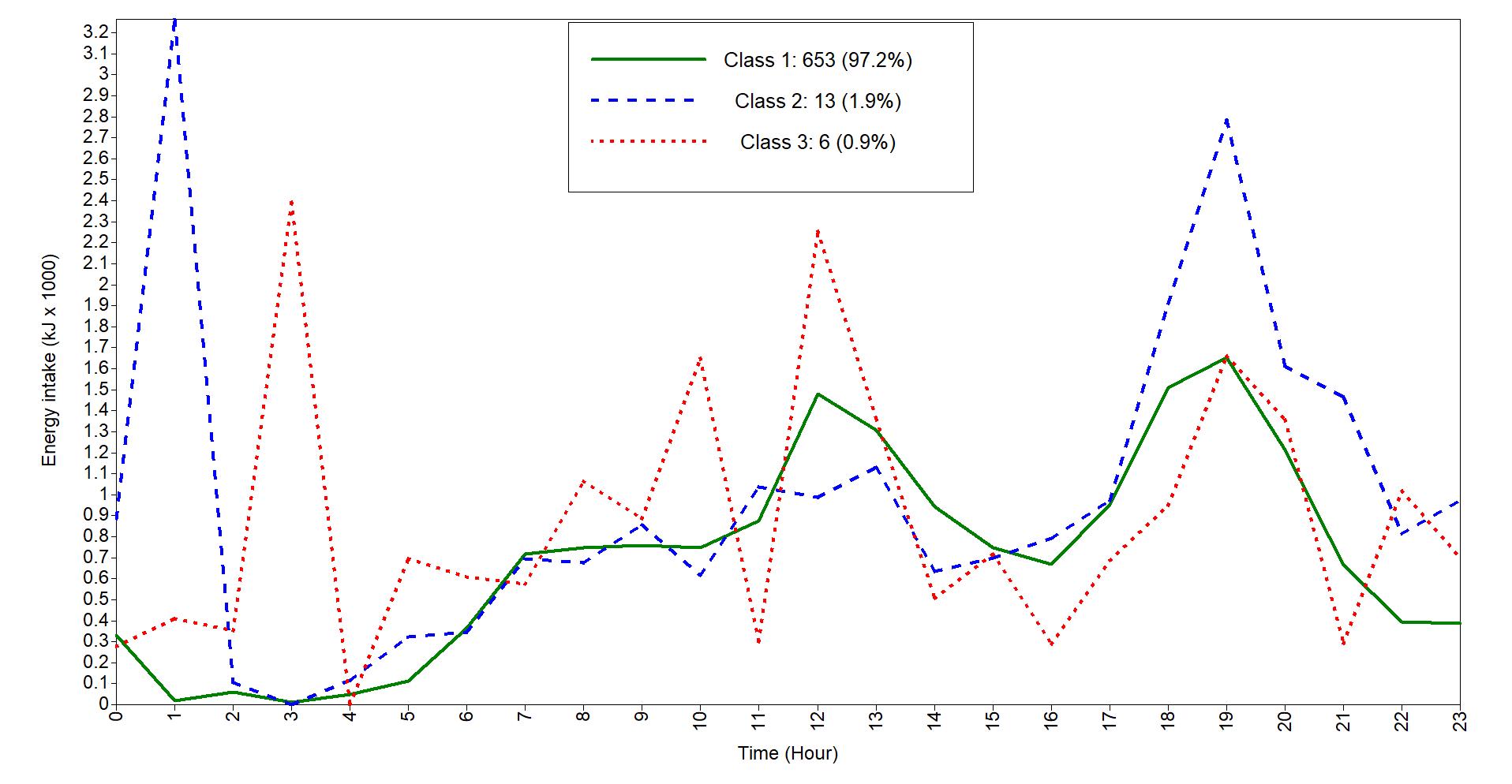


Figure 1: Three temporal eating pattern profiles identified using latent profile analysis based on continuous hourly energy intake among Australian adults (n = 672)
