# Supplementary material for: Identifying temporal eating patterns: a comparison of latent class analysis and dynamic time warping-based cluster analysis
Source: Am J Clin Nutr. 2026 Apr 15;123(6):101317. doi: 10.1016/j.ajcnut.2026.101317 (PMC13269345; doi:10.1016/j.ajcnut.2026.101317)
Supplement: Multimedia component 3 [file mmc3.docx]

Identifying temporal eating patterns: A comparison of latent class analysis and dynamic time warping-based cluster analysis: Beshada Rago Jima “Online Supplementary Material”

Python code for deriving temporal eating patterns

# =============Import important packages ======================

import pandas as pd

import numpy as np

import matplotlib.pyplot as plt

import seaborn as sns

from tslearn.preprocessing import TimeSeriesScalerMeanVariance

from tslearn.clustering import KernelKMeans

from tslearn.metrics import dtw, dtw_path

from collections import Counter

import pickle

import gc

from joblib import Parallel, delayed

import multiprocessing

# ============== Load your hourly average energy intake data =============

df = pd.read_stata("EI_Average.dta")

print(df['ParticipantId'].nunique())

#===============Ensure correct variable types =======================

df['Hour'] = df['Hour'].astype(int)

df['EI'] = df['EI'].astype(float)

# ============== Fill in missing hours (0–23) with zero EI for each participant ===

print(df['EI'].isna().sum())

all_hours = np.arange(24)

#df['EI'] = df['EI'].fillna(0)

participants = df['ParticipantId'].unique()

sequences = {}

for pid in participants:

sub = df[df['ParticipantId'] == pid].set_index('Hour')

energy = np.zeros(24)

for h in all_hours:

if h in sub.index:

energy[h] = sub.loc[h, 'EI']

hours = all_hours.astype(float)

multidim = np.stack((hours, energy), axis=1) # shape (24, 2)

sequences[pid] = multidim

ids = list(sequences.keys())

# ====Compute Modified (MDTW) distance matrix (Sakoe-Chiba radius = 3) ========

print("Computing MDTW_SakoeChiba.csv...")

sakoe_radius = 3

n = len(ids)

dtw_matrix = np.zeros((n, n))

def compute_mdtw(i, j):

s1 = sequences[ids[i]]

s2 = sequences[ids[j]]

path, dist = dtw_path(s1, s2, global_constraint="sakoe_chiba", sakoe_chiba_radius=sakoe_radius)

return (i, j, dist)

results = Parallel(n_jobs=multiprocessing.cpu_count())(

delayed(compute_mdtw)(i, j) for i in range(n) for j in range(i, n)

)

for i, j, dist in results:

dtw_matrix[i, j] = dist

dtw_matrix[j, i] = dist

dtw_df = pd.DataFrame(dtw_matrix, index=ids, columns=ids)

dtw_df.to_csv("MDTW_SakoeChiba.csv")

print("Saved MDTW_SakoeChiba.csv")

# ========== Standardize the energy intake values across time for each sample individually, so each participant's energy intake pattern is normalized (mean 0, std 1) ==========

X = np.array([sequences[pid] for pid in ids]) # shape (n_samples, 24, 2)

X_scaled = X.copy()

X_scaled[:, :, 1:] = TimeSeriesScalerMeanVariance().fit_transform(X[:, :, 1:])

#============== Evaluate Dunn and Silhouette Indexes==================

from sklearn.metrics import silhouette_score

from sklearn.metrics import pairwise_distances

def dunn_index(X, labels):

unique_labels = np.unique(labels)

distances = pairwise_distances(X)

intra_dists = [

np.max(distances[np.ix_(np.where(labels == lbl)[0], np.where(labels == lbl)[0])])

for lbl in unique_labels

]

inter_dists = [

np.min(distances[np.ix_(np.where(labels == i)[0], np.where(labels == j)[0])])

for i in unique_labels for j in unique_labels if i < j

]

return min(inter_dists) / max(intra_dists) if max(intra_dists) > 0 else 0

# ================Flatten input for distance-based evaluation================

X_flat = X_scaled[:, :, 1].squeeze() # shape: (n_samples, 24)

k_values = range(2, 7)

dunn_scores = []

silhouette_scores = []

print("Evaluating Dunn and Silhouette Indexes...\n")

for k in k_values:

print(f"Testing k = {k}...")

km = KernelKMeans(n_clusters=k, kernel="gak", kernel_params={"sigma": "auto"},

n_init=10, max_iter=100, random_state=42, verbose=False)

labels_k = km.fit_predict(X_scaled)

dunn = dunn_index(X_flat, labels_k)

silhouette = silhouette_score(X_flat, labels_k)

dunn_scores.append(dunn)

silhouette_scores.append(silhouette)

print(f" Dunn Index = {dunn:.4f}")

print(f" Silhouette Score = {silhouette:.4f}")

# ============Report all scores================

results_df = pd.DataFrame({

'k': list(k_values),

'Dunn Index': dunn_scores,

'Silhouette Score': silhouette_scores

})

print("\nClustering evaluation results:")

print(results_df.to_string(index=False))

# ============== Final Kernel K-Means clustering with k = 3 =================

def safe_kernel_kmeans(X, k, n_init=20, max_iter=5000, random_state=0):

for attempt in range(n_init):

km = KernelKMeans(n_clusters=k, kernel="gak", kernel_params={"sigma": "auto"},

max_iter=max_iter, random_state=random_state + attempt)

labels = km.fit_predict(X)

if len(set(labels)) == k:

return labels, km

print(f"Retrying... attempt {attempt+1}/{n_init} (empty cluster found)")

raise ValueError("Kernel K-Means failed to converge without empty clusters.")

k = 3

print(f"\nRunning final Kernel K-Means clustering for k={k}...")

labels, gak_km = safe_kernel_kmeans(X_scaled, k=k, n_init=20, max_iter=5000)

# ================ Organize participants into clusters =====================

clusters = {label: [] for label in range(k)}

for pid, label in zip(ids, labels):

clusters[label].append(pid)

# ================ Detect peak and second peak hours per participant =========

peak_times = {label: [] for label in clusters}

second_peak_times = {label: [] for label in clusters}

for label in clusters:

for pid in clusters[label]:

seq = sequences[pid] # (24, 2): [hour, EI]

ei_sorted_idx = np.argsort(seq[:, 1])[::-1]

peak_times[label].append(seq[ei_sorted_idx[0], 0])

if len(ei_sorted_idx) > 1:

second_peak_times[label].append(seq[ei_sorted_idx[1], 0])

# ================= Label remapping (if needed) =====================

label_mapping = {i: i for i in range(k)} # no remapping here

remapped_labels = np.array([label_mapping[label] for label in labels])

line_styles = {

0: ('green', '-'),

1: ('blue', '--'),

2: ('red', ':')

}

# =========== Plot 1: Cluster Centroids ===========================

plt.figure(figsize=(10, 5))

sns.set(style="white")

for label in sorted(np.unique(remapped_labels)):

cluster_sequences = [sequences[ids[i]][:, 1] for i in range(len(ids)) if remapped_labels[i] == label]

if not cluster_sequences:

continue

centroid = np.mean(cluster_sequences, axis=0)

n = len(cluster_sequences)

pct = (n / len(remapped_labels)) * 100

color, style = line_styles[label]

plt.plot(range(24), centroid, color=color, linestyle=style, linewidth=2,

label=f'Cluster {label + 1}: {n} ({pct:.1f}%)')

plt.title('Cluster Centroids')

plt.xlabel('Time (Hours)')

plt.ylabel('Energy Intake (kJ)')

plt.legend()

plt.grid(False)

plt.xticks(range(24))

plt.ylim(bottom=0)

plt.xlim(left=0)

plt.tight_layout()

plt.savefig('cluster_centroids.png', dpi=300)

plt.show()

# ================Plot 2: Mean ± 1 SD ==========================

plt.figure(figsize=(10, 5))

sns.set(style="white")

for label in sorted(np.unique(remapped_labels)):

cluster_sequences = np.array([sequences[ids[i]][:, 1] for i in range(len(ids)) if remapped_labels[i] == label])

if cluster_sequences.ndim != 2:

continue

mean_series = np.mean(cluster_sequences, axis=0)

std_series = np.std(cluster_sequences, axis=0)

color, style = line_styles[label]

n = cluster_sequences.shape[0]

pct = (n / len(remapped_labels)) * 100

plt.plot(range(24), mean_series, color=color, linestyle=style, linewidth=2,

label=f'Cluster {label + 1}: {n} ({pct:.1f}%)')

plt.fill_between(range(24), mean_series - std_series, mean_series + std_series, color=color, alpha=0.2)

plt.xlabel('Time (Hours)')

plt.ylabel('Energy Intake (kJ)')

plt.legend()

plt.grid(False)

plt.xticks(range(24))

# Set y-axis ticks every 250 kJ

max_y = plt.ylim()[1] # get current max y limit after plotting lines

plt.ylim(bottom=0)

plt.xlim(left=0)

plt.tight_layout()

plt.savefig('Mean energy intake +1 SD.png', dpi=300)

plt.show()

#==============Mean energy intake without SD ===========================

plt.figure(figsize=(10, 5))

sns.set(style="white")

for label in sorted(np.unique(remapped_labels)):

cluster_sequences = np.array([sequences[ids[i]][:, 1] for i in range(len(ids)) if remapped_labels[i] == label])

if cluster_sequences.ndim != 2:

continue

mean_series = np.mean(cluster_sequences, axis=0)

color, style = line_styles[label]

n = cluster_sequences.shape[0]

pct = (n / len(remapped_labels)) * 100

plt.plot(range(24), mean_series, color=color, linestyle=style, linewidth=2,

label=f'Cluster {label + 1}: {n} ({pct:.1f}%)')

#plt.title('Mean Energy Intake')

plt.xlabel('Time (Hours)')

plt.ylabel('Energy Intake (kJ)')

plt.legend()

plt.grid(False)

plt.xticks(range(24))

max_y = plt.ylim()[1] # get current max y limit after plotting lines

plt.yticks(np.arange(0, max_y + 250, 250))

plt.ylim(bottom=0)

plt.xlim(left=0)

plt.tight_layout()

plt.savefig('Mean EI without SD.png', dpi=300)

plt.show()

# ================ Time of the largest energy intake per Cluster ===============

plt.figure(figsize=(10, 5))

sns.set(style="white")

remapped_peak_times = {label_mapping[k]: v for k, v in peak_times.items()}

total_participants = sum(len(v) for v in remapped_peak_times.values())

for label in sorted(remapped_peak_times):

values = remapped_peak_times[label]

if not values:

continue

count = Counter(values)

unique_times = sorted(set(values))

freq = [count.get(i, 0) / len(values) for i in unique_times]

n = len(values)

pct = (n / total_participants) * 100

color, style = line_styles[label]

plt.plot(unique_times, freq, color=color, linestyle=style, linewidth=2,

label=f'Cluster {label + 1}: {n} ({pct:.1f}%)')

plt.xlabel('Time (Hours)')

plt.ylabel('Relative Frequency')

#plt.title('Time of Largest Energy Intake per Cluster')

plt.legend()

plt.grid(False)

plt.xticks(range(24))

plt.ylim(bottom=0)

plt.xlim(left=0)

plt.tight_layout()

plt.savefig('Time_of_largest_EI_kernel.png', dpi=300)

plt.show()

# ================= Time of second largest energy intake per Cluster ==========

plt.figure(figsize=(10, 5))

sns.set(style="white")

remapped_second_peak_times = {label_mapping[k]: v for k, v in second_peak_times.items()}

total_participants = sum(len(v) for v in remapped_second_peak_times.values())

for label in sorted(remapped_second_peak_times):

values = remapped_second_peak_times[label]

if not values:

continue

count = Counter(values)

unique_times = sorted(count.keys())

freq = [count[t] / len(values) for t in unique_times]

n = len(values)

pct = (n / total_participants) * 100

color, style = line_styles[label]

plt.plot(unique_times, freq, color=color, linestyle=style, linewidth=2,

label=f'Cluster {label + 1}: {n} ({pct:.1f}%)')

plt.xlabel('Time (Hours)')

plt.ylabel('Relative Frequency')

#plt.title('Time of Second Largest Energy Intake per Cluster')

plt.legend()

plt.grid(False)

plt.xticks(range(24))

plt.ylim(bottom=0)

plt.xlim(left=0)

plt.tight_layout()

plt.savefig('Time_of_second_largest_EI_kernel.png', dpi=300)

plt.show()

# ================ Median + IQR ============================

plt.figure(figsize=(10, 5))

sns.set(style="white")

plotted_any = False

for label in sorted(np.unique(remapped_labels)):

cluster_sequences = np.array([sequences[ids[i]][:, 1] for i in range(len(ids)) if remapped_labels[i] == label])

if cluster_sequences.ndim != 2:

continue

median_series = np.median(cluster_sequences, axis=0)

q25 = np.percentile(cluster_sequences, 25, axis=0)

q75 = np.percentile(cluster_sequences, 75, axis=0)

color, style = line_styles[label]

n = cluster_sequences.shape[0]

pct = (n / len(remapped_labels)) * 100

plt.plot(range(24), median_series, color=color, linestyle=style, linewidth=2,

label=f'Cluster {label + 1}: {n} ({pct:.1f}%)')

plt.fill_between(range(24), q25, q75, color=color, alpha=0.2)

plotted_any = True

if not plotted_any:

print("No valid cluster sequences to plot. Check your input data.")

plt.title('Median Energy Intake and IQR per Cluster')

plt.xlabel('Time (Hours)')

plt.ylabel('Energy Intake (kJ)')

plt.legend()

plt.grid(False)

plt.xticks(range(24))

plt.ylim(bottom=0)

plt.xlim(left=0)

plt.tight_layout()

plt.savefig('Median energy intake.png', dpi=300)

plt.show()

# === Save remapped cluster labels (+1) to Stata file ===

df_stata = pd.DataFrame({

'ParticipantId': ids,

'Cluster': remapped_labels + 1

})

print(df['ParticipantId'].nunique())

df_stata.to_stata('Clusters_kernel_kmeans.dta', write_index=False)

gc.collect()
